# Effectiveness of Sectioning Method and Filling Materials on Roughness and Cell Attachments in Root Resection Procedure

**DOI:** 10.1055/s-0044-1788319

**Published:** 2024-07-19

**Authors:** Tarek Ashi, Naji Kharouf, Olivier Etienne, Bérangère Cournault, Pierre Klienkoff, Varvara Gribova, Youssef Haikel

**Affiliations:** 1 Department of Biomaterials and Bioengineering, University of Strasbourg, INSERM UMR_S 1121, Strasbourg, France; 2 Department of Endodontics and Conservative Dentistry, Faculty of Dental Medicine, University of Strasbourg, Strasbourg, France; 3 Department of Prosthodontics, Faculty of Dental Medicine, University of Strasbourg, Strasbourg, France; 4 Pôle de Médecine et Chirurgie Bucco-Dentaire, Hôpital Civil, Hôpitaux Universitaire de Strasbourg, Strasbourg, France

**Keywords:** apicoectomy, bioceramic, cell attachment, roughness, tooth resection

## Abstract

**Objectives** The purpose of the present study was to investigate the created roughness and cell attachment of intact teeth (C), obturated teeth with bioceramic (BR), or epoxy resin (AH) after root resection using piezoelectric ultrasonic and carbide bur.

**Materials and Methods** Three groups of first mandibular premolars were used in the present study: control group (without any preparation or obturation) (C); second group was obturated with an epoxy resin sealer (AH, AH Plus Jet); and finally, the third one was obturated with a bioceramic sealer (BR, BioRoot RCS). All teeth were incubated for 4 months at 37°C. After that, the samples were sectioned using tungsten carbide bur or piezoelectric ultrasonic. Roughness and then cell attachment of periodontal ligament cells on the sectioned surfaces were investigated by profilometer and confocal microscope, respectively.

**Statistical Analysis** Data were statistically analyzed using one-way analysis of variance.

**Results** After root resection, no significant difference was found between the roughness among the three groups sectioned using the piezoelectric technique ( *p* > 0.05). In contrast, concerning the sectioned samples by burs, C demonstrated a rougher surface compared with BR ( *p* < 0.05). There was a significant higher cell attachment in BR compared with AH in the piezoelectric groups ( *p* = 0.047), while no statistically significant difference was found between the groups sectioned with bur ( *p* > 0.05).

**Conclusion** Dentists are now focused on the use of calcium silicate-based sealers due to their bioactivity. The present study advises dentists to use bioceramic sealer which could improve the dentin characteristics which ameliorate the cell attachment.

## Introduction

Surgical endodontic therapy becomes the treatment of choice when teeth exhibit inadequate responses to nonsurgical treatments, retreatment, or when nonsurgical methods prove ineffective. 1 The goal of endodontic surgery, also known as apicoectomy, is to maintain the tooth and eliminate periradicular pathosis, thereby restoring the health and functionality of the tooth's periodontium. 2

Root-end resection, retrograde cavity preparation, and root-end filling are essential parts of endodontic microsurgery procedure. Apical anatomy studies suggest that during surgical procedures, it is recommended to resect the root apex by 2 to 3 mm. This ensures the removal of the majority of unprepared and unfilled accessory canals, thereby effectively eliminating potential reservoirs of pathogens. 3 4 Following these steps, an ideal material for root-end filling should be applied. This material should possess biocompatibility, strong adhesion to tooth structure, dimensional stability, resistance to dissolution, antibacterial properties, and ease of use. 5 6 7 The objective is to create a hermetic seal at the apex, preventing the entry of microorganisms into the root canal. 8 9

Endodontic microsurgery provides precise and predictable outcomes as well as eliminates the assumptions associated with specific instruments. 10 When performing tooth resection, it is crucial to minimize surface roughness and the occurrence of microfractures, as they can significantly influence the success or failure of retrograde treatment. Therefore, the size, shape, and material of the drill are important factors that affect the results of surface quality. 11

Ultrasonic devices have become increasingly gained popularity for preparing root-end cavities, surpassing the use of carbide or diamond burs. 12 Various studies have indicated that instruments such as diamond-coated tips and ultrasound devices are commonly used for cavity preparation. 13 14 15 Other studies comparing different instruments and techniques for root-end resection, including steel fissure bur, tungsten carbide bur, Zekrya bur, diamond-tip piezoelectric, ultrasonic device, and Er:YAG laser, have consistently concluded that the tungsten carbide bur can be considered the most suitable method for apical resection. 16 17

To achieve optimal root resection, it is crucial to employ a suitable method and instruments such as carbide bur 17 or piezoelectric technique 18 that results in a regular and smooth apical surface with minimal cracks to ensure a successful treatment outcome. 19 Achieving a smooth surface is important in reducing the exposure of dentinal tubules on the resected root surface and minimizing apical leakage. Rough and irregular surfaces can act as irritants, accumulate debris, and potentially stimulate resorption during the healing process. Furthermore, creating an appropriate surface condition is crucial to facilitate favorable cellular attachment and promote optimal healing outcomes by synthesizing new matrix components. 20 21 In ideal healing responses, the attachment of periodontal ligament (PDL) fibroblasts to the resected tooth structure is critical. The ability of these cells to attach to the root surface plays a significant role in promoting proper healing and favorable treatment outcomes. 22

Previous studies shown the importance of bioactive endodontic sealers in root canal treatment, particularly in remineralization and mineral infiltration as well as deposition into the dentinal tubules, compared with traditional epoxy resin sealer. 23 24 25 Moreover, these bioceramic sealers could infiltrate into dentinal tubules and fill them. In addition, their chemical composition includes calcium silicate elements that have biological and physicochemical properties that affect the dentin structure by the release of Ca ^2+^ and the alkaline pH. 23 Yoo et al demonstrated that the use of calcium silicate-based materials could lead to the deposition of minerals within the dental tubules after 4 months of age. 26 However, the impact of this mineral deposition on the quality of resected surface, including surface roughness and cell attachment, has not been investigated in the literature.

The purpose of this *in vitro* study is to evaluate the change in roughness and cell attachment in intact, and obturated teeth with bioceramic or epoxy resin sealers following sectioning procedure using piezoelectric or bur techniques. The null hypothesis is that the obturation material or the sectioning technique has no effect on cell attachment and roughness values.

## Materials and Methods

### Teeth Preparation

Ninety-six single-rooted, single-canal first mandibular premolars with complete development, extracted for periodontal or orthodontic reasons, were included in the present *in vitro* study. The ethical protocol number: CE-2024-50 was obtained from the “Comité d'Ethique des Facultés de Médecine, d'Odontologie, de Pharmacie, des Ecoles d'Infirmières, de Kinésithérapie, de Sages-Femmes et des Hôpitaux Universitaires de Strasbourg” in order to use the extracted teeth in research studies.

The samples were randomly divided into three equal groups ( *n* = 32) as follows:

Group 1 “control group C”: Intact teeth with no endodontic/restorative preparation.
Group 2: Teeth prepared and obturated with AH Plus Jet “AH” (Dentsply DeTrey GmbH, Konstanz, Germany).
Group 3: Teeth prepared and obturated with BioRoot RCS “BR” (Septodont, Louisville, Colorado, United States).

All the cusps were flattened by polishing with a silicon carbide paper (120-grit) using a polishing machine (Escil, Chassieu, France) to standardize the length of root canal at 20 ± 1 mm. For Groups 2 and 3, access cavities were prepared by using diamond burs and ultrasonic tips under loops (Eighteeth, Changzhou City, Jiangsu Province, China). A 10-K-file was used to the manual scouting and the working length was determined to be 0.5 mm from the apical foramen. The root canals were prepared by using Ni-Ti MG3 Bleu sequence up to 25/06 (Dental Perfect, Shenzhen City, Guangdong Province, China) powered by an endodontic motor (Dental Perfect). A single operator, an expert endodontist, prepared all the teeth. Each canal was irrigated with 5 mL of 17% ethylenediaminetetraacetic acid (EDTA) (Septodont) and 5 mL of 3% of sodium hypochlorite (Septodont) an activated with the “Ultra Dancer” device (Dental Perfect). After drying with paper points, the canals were obturated using single cone technique with AH in Group 2 and BR and Group 3.

All teeth (Groups1–3) were stored in phosphate-buffered saline (PBS ×10, Dominique Dutscher, Bernolsheim, France) for 4 months at 37°C in an incubator.

### Apicoectomy Procedure

**Fig. 1:**
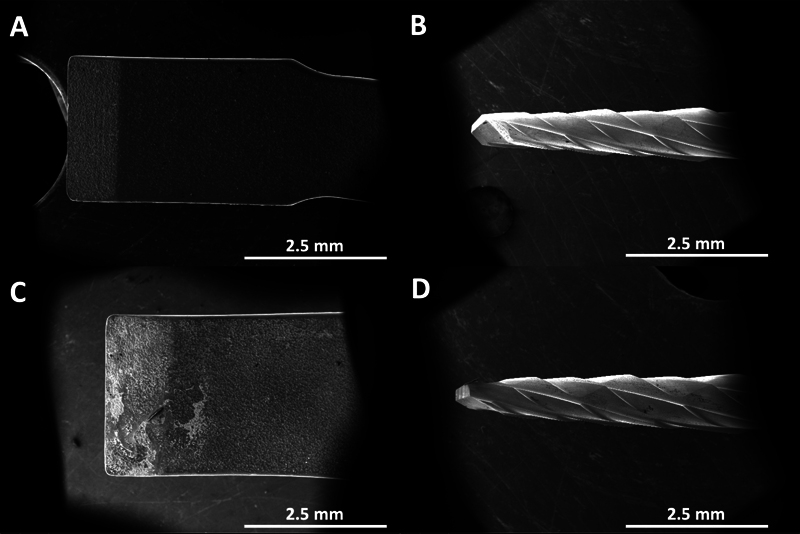
Scanning electron microscope images demonstrated: ( **A** ) new piezoelectric ultrasonic bone surgery; ( **B** ) new tungsten carbide bone cutter bur; ( **C** ) used piezoelectric ultrasonic bone surgery for five times; and ( **D** ) used tungsten carbide bone cutter bur for five times.

After aging for 4 months, each tooth sample was surrounded by melted wax to simulate 2- to 3-mm-thick PDL except for the last apical 3 mm. The teeth were then embedded in acrylic self-curing resin (OrthocrylEQ, Dentaurum, Ispringen, Germany), leaving the apical part (3 mm) of the root exposed. Subsequently, the wax was replaced with a silicon-based impression material, following the method outlined in a previous study. 27 To ensure blinded evaluation, all teeth were assigned codes ranging from 1 to 96. the sectioning of the teeth was performed perpendicular to their longitudinal axis, 3 mm from the apex, under cooling water. This procedure was carried out by same operator using two distinct techniques for each group, creating six subgroups ( *n* = 16) as follows:

Groups C_P, AH_P, and BR_P: piezoelectric ultrasonic bone surgery system (NSK, VarioSurg3, Tokyo, Japan) in endo mode without burst coupled using a piezoelectric ultrasonic insert (Model: US3, Woodpecker Medical Instrument Co, Guangxi, China) ( Fig. 1A ).
Groups C_B, AH_B, and BR_B: tungsten carbide bone cutter bur (H254LE.314.012, Komet, Paris, France) at a speed of 150,000 RPM ( Fig. 1B ).

Each bur or insert was used for five procedures before being replaced with a new one.

### Surface Roughness

All samples were examined using an optical profilometer (InfiniteFocus SL, Bruker Alicona, Graz, Austria) at a magnification of ×10, with a lateral resolution of 5 µm and a vertical resolution of 1 µm. The arithmetic mean roughness (Ra) of each sample was determined across five distinct areas, following to ISO 4287 guidelines. Each Ra measurement was conducted along a profile extending at least 12.5 mm in length, following a sawtooth pattern perpendicular to the surface relief, and utilized a cutoff filter (Lc) of 2,500 µm.

### Cell Attachment

**Fig. 2:**
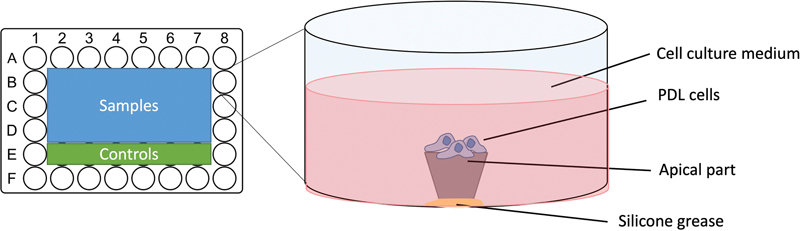
Schematic graph demonstrating human periodontal ligament (PDL) cells seeding procedure onto the sectioned surface.

Eighteen resected samples ( *n* = 3 for each subgroup) were utilized. The apices underwent sterilization using PSM-UV (ADS Laminaire, Aulnay-sous-bois, France) for 60 minutes. Human PDL cells were extracted from alveolar ligament of an extracted tooth with the donor's consent. These cells were cultured in Dulbecco's modified Eagle medium (Dominique Dutscher) high glucose supplemented with 10% fetal bovine serum and 1% penicillin–streptomycin, maintained at 37°C in a 5% CO _2_ atmosphere. Routine passaging was done using trypsin EDTA, and the cells were used between passages 3 and 5. The apices were placed in a 48-well plate, held upright by the apical part using silicone grease (Beckman Coulter, Inc., Brea, California, United States) to expose the sectioned surface ( Fig. 2 ). For seeding, 40,000 cells in 400 µL of medium were added to each well and incubated for 48 hours at 37°C.

### Confocal Observation

After 48 hours of incubation at 37°C, the culture medium was removed, and cells were fixed with 4% paraformaldehyde (Euromedex, Souffelweyersheim, France) in PBS for 30 minutes. Subsequently, the fixed cells were incubated with phalloidin 1/500 and DAPI 1/1,000 in PBS for 10 minutes, followed by a rinse in PBS. Observations were conducted using Zeiss LSM 710 confocal microscope (Zeiss, Heidelberg, Germany) at a ×20 magnification, employing two wavelengths of 405 and 561 nm.

### Scanning Electron Microscopy

Initially, a new and a used sectioning instrument from each group were examined using a scanning electron microscope (SEM) (FEI Company, Eindhoven, The Netherlands, 10 kV) at a magnification of ×30 with a working distance of 10 mm. Additionally, to observe the structural changes and dentinal tubules, three randomly samples of BR were examined after aging for 4 months. For this evaluation, the samples were fractured longitudinally in the middle and horizontally (3 mm from the sectioned surface), mounted on SEM stubs, and sputter-coated with a gold–palladium alloy (20/80 weight %) using a sputtering device (Technics, California, United States), and subjected to SEM observation.

### Statistical Analysis

Data were statistically analyzed with SigmaPlot release 11.0 (Systat Software, Inc., San Jose, California, United States). One-way analysis of variance was used to determine whether significant differences existed between the groups for roughness and cell attachment tests. The statistical significance level was set at α = 0.05.

## Results

### Roughness

**Fig. 3:**
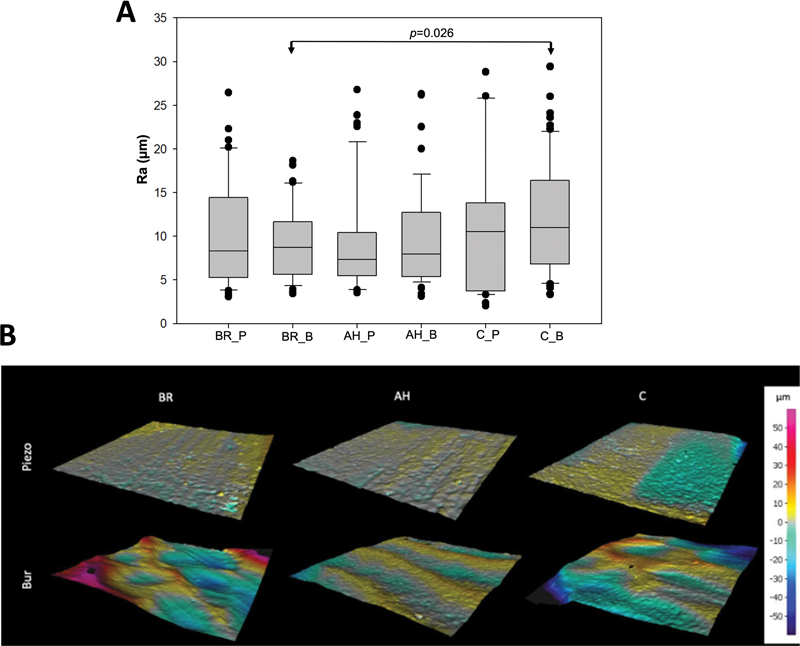
( **A** ) Schematic graph demonstrated the means and standard deviations (Ra “µm”) of all analyzed surfaces using a profilometer. C: Intact teeth with no endodontic/restorative preparation; AH: teeth prepared and obturated with AH Plus Jet; BR: teeth prepared and obturated with BioRoot RCS; P: piezoelectric; and B: bur (* *p* < 0.05). ( **B** ) Micrographs taken by a profilometer demonstrated the roughness of sample surfaces. C: Intact teeth with no endodontic/restorative preparation; AH: teeth prepared and obturated with AH Plus Jet; and BR: teeth prepared and obturated with BioRoot RCS.

No statistically significant difference in surface roughness between BR samples and those prepared using the piezoelectric techniques ( *p* > 0.05) within each group. Moreover, no significant difference was found between the three groups sectioned by using piezoelectric technique ( *p* > 0.05). In contrast, samples sectioned with burs showed that group C_B had a rougher surface compared with BR_B ( *p* < 0.05). However, there were no statistically significant differences between C_B and AH_B or between AH_B and BR_B ( *p* > 0.05) ( Fig. 3 ).

### Cell Attachment

**Fig. 4:**
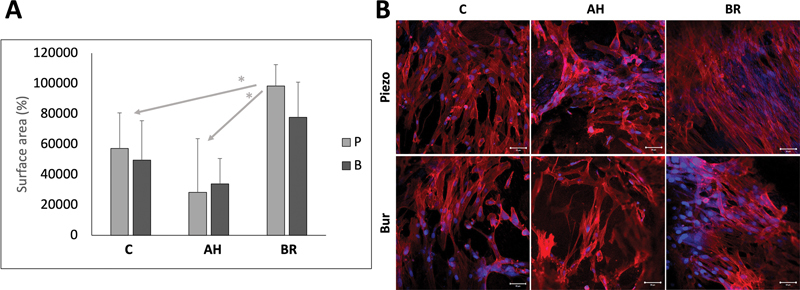
( **A** ) Percentages of covered area by cells using confocal microscope images. C: Intact teeth with no endodontic/restorative preparation; AH: teeth prepared and obturated with AH Plus Jet; BR: teeth prepared and obturated with BioRoot RCS; P: piezoelectric; and B: bur. ( **B** ) Confocal microscopy images demonstrating cell attachment onto the different tested surfaces after 48 hours of incubation at 37°C. C: Intact teeth with no endodontic/restorative preparation; AH: teeth prepared and obturated with AH Plus Jet; and BR: teeth prepared and obturated with BioRoot RCS.

No significant differences were found between piezoelectric and bur in each group ( *p* > 0.05). In contrast, comparing the cell attachment between the three groups (C, AH, and BR), there was a significantly higher cell attachment in BR compared with AH and C in piezoelectric groups ( *p* = 0.047), while no statistically significant difference was found between the groups sectioned with bur ( *p* > 0.05) ( Fig. 4 ).

## Discussion

The aim of endodontic microsurgery is to preserve teeth that have not responded to the traditional orthograde treatment or retreatment. Various factors can affect the healing of the resected tooth, including the formation of cracks during apicoectomy and root-end preparation, 16 retrograde cavity design, 28 retrograde filling materials, 29 and the homogeneity and roughness of the sectioned surfaces. 19

In addition, certain materials used in root canal obturation may exert biological, 30 mechanical, 28 31 and physicochemical 32 effects on the tooth's canal system, including dentinal tubules and overall structure. BioRoot RCS was used in the present study due to its previously observed bioactivity such as Ca ^2+^ release, pH changes, and the remineralization process. 23 Conversely, epoxy resin materials such as AH Plus Jet ^®^ have not shown calcium ion release, 33 or the lower pH levels associated with bioceramic materials 25 which could not promote the remineralization process.

In the present study, two root-resection instruments were compared among three teeth groups: intact teeth, teeth obturated with bioceramic, and teeth obturated with epoxy resin materials. The utilization of piezoelectric and tungsten carbide burs was grounded in their recognition as the foremost techniques for ensuring a safe and effective endodontic microsurgical treatment. 17 18 The selection of the intact teeth as a control group was strategic given that one indication of the apicoectomy process is the inaccessibility of orthograde treatment, 34 leaving the apical portion undisturbed. Additionally, using intact teeth without any chemical treatment or obturation material effects on their dentin serves as baseline for comparison with the treated groups (AH and BR).

Profilometry was used to analyze surface roughness postsectioning. No significant differences were detected between the piezoelectric and bur preparations within each group ( *p* > 0.05). In accordance to our study, Ekici et al 18 demonstrated no significant difference between the roughness of surfaces resected by piezoelectric and carbide bur techniques. However, they noted that the carbide bur method achieved similar roughness levels with an advantageous less time than the piezoelectric technique. Notably, the C_B group exhibited rougher surface compared with BR_B ( *p* < 0.05), necessitating a partial rejection of the null hypothesis. All teeth were sectioned at a 90-degree angle to minimize exposed dentinal tubules and surface roughness, thereby reducing contamination risk. 35 A uniform and smoother surface is preferable for promoting healing, minimizing contamination and facilitating the application of root-end filling materials. 18 36

**Fig. 5:**
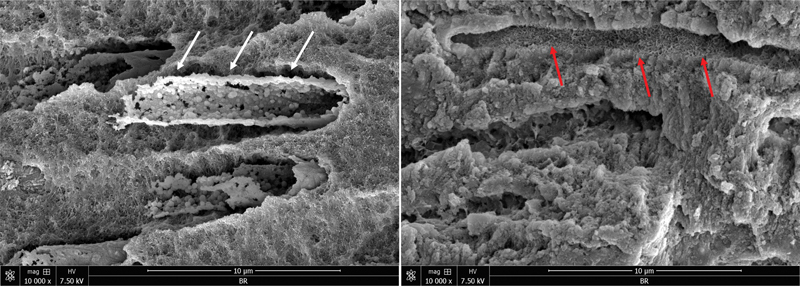
Scanning electron microscope images demonstrate the mineral deposition (red arrows) and calcium silicate infiltration (white arrows) of BioRoot RCS into dentinal tubules after 4 months of aging period in phosphate-buffered saline at 37°C.

Cell attachment was evaluated using a confocal microscope after 48 hours of incubation at 37°C, then percentage of the area covered by the cells was quantified. No significant differences were found between piezoelectric and bur techniques within each group ( *p* > 0.05) which is in accordance with the roughness results. In contrast, when the cell attachment was compared between the three groups (C, AH, and BR), there was a significantly higher cell attachment in BR compared with C and AH in the piezoelectric groups ( *p* = 0.047), while no significant difference was found between the groups sectioned with bur ( *p* > 0.05). Regardless of the sectioning technique, when the results of piezoelectric and bur in each group were cumulated, BR demonstrated higher cell attachment compared with AH and C ( *p* = 0.002). This could be attributed to the cytotoxicity reported in epoxy resin materials and the better biocompatibility of bioceramic materials with dental cells. 37 These findings could also be related to the fact that bioceramic materials have bioactive properties as described previously 24 25 and could create an optimal environment for intratubular mineralization after 4 months of obturation. 26 Therefore, it could be hypothesized that the realization of Ca ^2+^ ions from bioceramic materials could increase the amount of calcium ions in root dentin as well as the mineral deposition in dentinal tubules (filled dentinal tubules) ( Fig. 5 ). The mineral deposition was also observed in a previous study where the authors used mineral trioxide aggregate and the samples were incubated in PBS during only 1 month. 38 Moreover, it is plausible that the released calcium ions could play an important role to promote and enhance the cell attachment. 39 The primary objective postapicoectomy is the periapical regeneration through the deposition of bone, PDL, and cementum onto the resected surface. 40

This *in vitro* study has several limitations. The methodology used to simulate the PDL and the application of PBS during all the storage periods could be useful to avoid teeth desiccation. However, the study remains an *in vitro* study, and as such, it may not accurately represent the attachment of only PDL cells to dentinal surfaces *in vivo* conditions. In addition, more samples could provide a more comprehensive understanding of the cell attachment rates. Further research should be performed to investigate the mechanical changes in dentin structure due to the use of bioceramic as an initial filling material. The releasing of calcium ions could modify the morphology and the mechanical properties of dentin structure; thus, the mechanical properties of this structure should be studied in further research by using microhardness, flexural, and compression strength tests. It would also be beneficial to examine the progression of mineral deposition within the dentinal tubules at various aging periods following bioceramic obturation.

## Conclusion

Within the limitations of the present *in vitro* study, teeth obturated with bioceramic material could play a positive role on the cell attachment rate compared with epoxy resin sealer after an apical resection using carbide bur. No remarkable differences were found concerning the roughness of the different techniques or obturations materials. Further *in vivo* and *in vitro* studies should be performed to confirm these findings.

## References

[1] Cohen S Burns R C Pathways of the Pulp 6th ed. St. Louis Mosby 1994 753

[2] Lin C P Chen Y J Lee Y L Effects of root-end filling materials and eugenol on mitochondrial dehydrogenase activity and cytotoxicity to human periodontal ligament fibroblasts J Biomed Mater Res B Appl Biomater 2004 71 02 429 440 15389508 10.1002/jbm.b.30107

[3] Hsu Y Y Kim S The resected root surface. The issue of canal isthmuses Dent Clin North Am 1997 41 03 529 540 9248689

[4] Morfis A Sylaras S N Georgopoulou M Kernani M Prountzos F Study of the apices of human permanent teeth with the use of a scanning electron microscope Oral Surg Oral Med Oral Pathol 1994 77 02 172 176 8139836 10.1016/0030-4220(94)90281-x

[5] Haapasalo M Endal U Zandi H Coil J M Eradication of endodontic infection by instrumentation and irrigation solutions Endod Topics 2005 10 01 77 102

[6] Park E Shen Y Haapasalo M Irrigation of the apical root canal: irrigation of the apical root canal Endod Topics 2012 27 01 54 73

[7] Sundqvist G Figdor D Persson S Sjögren U Microbiologic analysis of teeth with failed endodontic treatment and the outcome of conservative re-treatment Oral Surg Oral Med Oral Pathol Oral Radiol Endod 1998 85 01 86 93 9474621 10.1016/s1079-2104(98)90404-8

[8] Keleş A Alcin H Kamalak A Versiani M A Micro-CT evaluation of root filling quality in oval-shaped canals Int Endod J 2014 47 12 1177 1184 24527697 10.1111/iej.12269

[9] Mohammadi Z Abbott P V The properties and applications of chlorhexidine in endodontics Int Endod J 2009 42 04 288 302 19220510 10.1111/j.1365-2591.2008.01540.x

[10] Kim S Kratchman S Modern endodontic surgery concepts and practice: a review J Endod 2006 32 07 601 623 16793466 10.1016/j.joen.2005.12.010

[11] Bernardes R A Húngaro Duarte M A Vivan R R Baldi J V Vasconcelos B C Bramante C M Scanning electronic microscopy analysis of the apical surface after of root-end resection with different methods Scanning 2015 37 02 126 130 25652816 10.1002/sca.21188

[12] Abedi H R Van Mierlo B L Wilder-Smith P Torabinejad M Effects of ultrasonic root-end cavity preparation on the root apex Oral Surg Oral Med Oral Pathol Oral Radiol Endod 1995 80 02 207 213 7552887 10.1016/s1079-2104(05)80204-5

[13] Brent P D Morgan L A Marshall J G Baumgartner J C Evaluation of diamond-coated ultrasonic instruments for root-end preparation J Endod 1999 25 10 672 675 10687526 10.1016/S0099-2399(99)80353-7

[14] Kwak S W Moon Y M Yoo Y J Baek S H Lee W Kim H C Cutting efficiency of apical preparation using ultrasonic tips with microprojections: confocal laser scanning microscopy study Restor Dent Endod 2014 39 04 276 281 25383346 10.5395/rde.2014.39.4.276 PMC4223097

[15] Tawil P Z Periapical microsurgery: can ultrasonic root-end preparations clinically create or propagate dentinal defects? J Endod 2016 42 10 1472 1475 27576210 10.1016/j.joen.2016.07.016

[16] Ayranci F Ayranci L B Arslan H Omezli M M Topcu M C Assessment of root surfaces of apicected teeth: a scanning electron microscopy evaluation Niger J Clin Pract 2015 18 02 198 202 25665992 10.4103/1119-3077.151041

[17] Bernardes R A de Souza Junior J V Duarte M A de Moraes I G Bramante C M Ultrasonic chemical vapor deposition-coated tip versus high- and low-speed carbide burs for apicoectomy: time required for resection and scanning electron microscopy analysis of the root-end surfaces J Endod 2009 35 02 265 268 19166787 10.1016/j.joen.2008.11.009

[18] Ekici Ö Aslantaş K Kanık Ö Keleş A Evaluation of surface roughness after root resection: an optical profilometer study Microsc Res Tech 2021 84 04 828 836 33491839 10.1002/jemt.23714

[19] Duarte M A Domingues R Matsumoto M A Padovan L E Kuga M C Evaluation of apical surface roughness after root resection: a scanning electron microscopic study Oral Surg Oral Med Oral Pathol Oral Radiol Endod 2007 104 06 e74 e76 17942334 10.1016/j.tripleo.2007.07.012

[20] Al-Nazhan S SEM observations of the attachment of human periodontal ligament fibroblasts to non-demineralized dentin surface in vitro Oral Surg Oral Med Oral Pathol Oral Radiol Endod 2004 97 03 393 397 15024366 10.1016/j.tripleo.2003.08.004

[21] Balto H Al-Nazhan S Attachment of human periodontal ligament fibroblasts to 3 different root-end filling materials: scanning electron microscope observation Oral Surg Oral Med Oral Pathol Oral Radiol Endod 2003 95 02 222 227 12582364 10.1067/moe.2003.96

[22] Fujii T Iwai H Kowashi Y Matsuo A Yajima T Kato H [Connective tissue attachment to root surfaces in periodontal disease. Initial attachment of human gingival fibroblasts] Nippon Shishubyo Gakkai Kaishi 1989 31 01 176 183 2700356 10.2329/perio.31.176

[23] Kharouf N Arntz Y Eid A Physicochemical and antibacterial properties of novel, premixed calcium silicate-based sealer compared to powder-liquid bioceramic sealer J Clin Med 2020 9 10 3096 32992852 10.3390/jcm9103096 PMC7600315

[24] Farrayeh A Akil S Eid A Effectiveness of two endodontic instruments in calcium silicate-based sealer retreatment Bioengineering (Basel) 2023 10 03 362 36978753 10.3390/bioengineering10030362 PMC10045724

[25] Kharouf N Sauro S Eid A Physicochemical and mechanical properties of premixed calcium silicate and resin sealers J Funct Biomater 2022 14 01 9 36662056 10.3390/jfb14010009 PMC9866383

[26] Yoo J S Chang S W Oh S R Bacterial entombment by intratubular mineralization following orthograde mineral trioxide aggregate obturation: a scanning electron microscopy study Int J Oral Sci 2014 6 04 227 232 25012869 10.1038/ijos.2014.30 PMC5153584

[27] Kharouf N Pedullà E Plotino G Stronger than ever: multifilament fiberglass posts boost maxillary premolar fracture resistance J Clin Med 2023 12 08 2975 37109310 10.3390/jcm12082975 PMC10143755

[28] Ashi T Richert R Mancino D Do the mechanical properties of calcium-silicate-based cements influence the stress distribution of different retrograde cavity preparations? Materials (Basel) 2023 16 08 3111 37109947 10.3390/ma16083111 PMC10145818

[29] Ashi T Mancino D Hardan L Physicochemical and antibacterial properties of bioactive retrograde filling materials Bioengineering (Basel) 2022 9 11 624 36354535 10.3390/bioengineering9110624 PMC9687475

[30] Hachem C E Chedid J CA Nehme W Physicochemical and antibacterial properties of conventional and two premixed root canal filling materials in primary teeth J Funct Biomater 2022 13 04 177 36278646 10.3390/jfb13040177 PMC9589963

[31] Kharouf N Sauro S Hardan L Haikel Y Mancino D Special issue “recent advances in biomaterials and dental disease” part I Bioengineering (Basel) 2023 10 01 55 36671627 10.3390/bioengineering10010055 PMC9854530

[32] Kharouf N Sauro S Hardan L Impacts of resveratrol and pyrogallol on physicochemical, mechanical and biological properties of epoxy-resin sealers Bioengineering (Basel) 2022 9 03 85 35324774 10.3390/bioengineering9030085 PMC8945518

[33] Chopra V Davis G Baysan A Physico-chemical properties of calcium-silicate vs. resin based sealers-a systematic review and meta-analysis of laboratory-based studies Materials (Basel) 2021 15 01 229 35009375 10.3390/ma15010229 PMC8745986

[34] Molven O Halse A Grung B Surgical management of endodontic failures: indications and treatment results Int Dent J 1991 41 01 33 42 2004837

[35] Vercellotti T De Paoli S Nevins M The piezoelectric bony window osteotomy and sinus membrane elevation: introduction of a new technique for simplification of the sinus augmentation procedure Int J Periodontics Restorative Dent 2001 21 06 561 567 11794567

[36] Camargo Villela Berbert F L de Faria-Júnior N B Tanomaru-Filho M An in vitro evaluation of apicoectomies and retropreparations using different methods Oral Surg Oral Med Oral Pathol Oral Radiol Endod 2010 110 04 e57 e63 10.1016/j.tripleo.2010.03.004 20573528

[37] Özdemir O Kopac T Cytotoxicity and biocompatibility of root canal sealers: a review on recent studies J Appl Biomater Funct Mater 2022 20 22808000221076325 10.1177/22808000221076325 35164598

[38] Park J H Kim H J Lee K W Yu M K Min K S Push-out bond strength and intratubular biomineralization of a hydraulic root-end filling material premixed with dimethyl sulfoxide as a vehicle Restor Dent Endod 2023 48 01 e8 36875809 10.5395/rde.2023.48.e8 PMC9982241

[39] Bae W J Chang S W Lee S I Kum K Y Bae K S Kim E C Human periodontal ligament cell response to a newly developed calcium phosphate-based root canal sealer J Endod 2010 36 10 1658 1663 20850672 10.1016/j.joen.2010.06.022

[40] Fayad M I Hawkinson R Daniel J Hao J The effect of CO2 laser irradiation on PDL cell attachment to resected root surfaces Oral Surg Oral Med Oral Pathol Oral Radiol Endod 2004 97 04 518 523 15088038 10.1016/j.tripleo.2003.10.028

